# Individual differences in thermoeffector function in the heat: morphological variations help determine effector activation

**DOI:** 10.1186/2046-7648-4-S1-A102

**Published:** 2015-09-14

**Authors:** Sean R Notley, Joonhee Park, Kyoko Tagami, Norikazu Ohnishi, Nigel AS Taylor

**Affiliations:** 1 Centre for Human and Applied Physiology, School of Medicine, University of Wollongong, Wollongong, Australia

## Introduction

It is possible that much of the inter-individual variability observed within human thermoeffector responses can be explained by differences in body morphology, specifically the ratio between surface area and mass. However, few have examined these relationships across a sufficiently wide range of body sizes, while controlling for the factors that can independently alter heat dissipation. This investigation was aimed at identifying the proportion of thermoeffector variability that could be explained on the basis of morphology within individuals of widely different surface-area-to-mass ratios, but of similar age, fitness and adiposity.

## Methods

Thermoeffector responses were examined in 36 males with pronounced difference in their surface area-to-mass ratio (range: 232.3-292.7 cm^2^.kg^-1^). Subjects completed two trials, both under temperate-dry conditions (28°C; 30% relative humidity). On separate days, participants completed 20 min of seated rest, then performed 45 min of steady-state, semi-recumbent cycling at a matched internal heat production rate (metabolic heat - external work) for each subject equal to ~135 W.m^-2 ^(trial one) or ~200 W.m^-2 ^(trial two), followed by a 20-min seated recovery. Deep-body and skin temperatures, whole-body sweat rate (change in body mass) and local sweat secretion (hand, forearm, upper back and forehead; ventilated capsules) and skin blood flow (forearm and finger; plethysmography) were measured (final 5 min of exercise). Multiple regression analyses were performed to evaluate the relationship between the change in mean body temperature (ΔT_b_) and surface-area-to-mass ratio on skin blood flow and sweating. An hierarchical model was employed, with ΔT_b _entered at the first step, and with the surface-area-to-mass ratio entered second. Changes in the coefficients of determination between those models were used to identify the proportion of thermoeffector variance that could be explained simply on the basis of differences in body morphology.

## Results

Surface-area-to-mass ratio was a significant predictor of the level of sweating and skin blood flow measured during trial one (~135 W.m^-2^; *P*<0.05), accounting for 22% of the variation in whole-body sweat rate, 21-25% in local-sweat rates, and between 27-40% of the skin blood flow responses. During trial two (~200 W.m^-2^), surface-area-to-mass ratio shared a significant relationship with whole-body sweat rate (*P*<0.05), explaining up to 65% of the variation among individuals, but did not demonstrate a significant relationship with either local-sweat secretion or skin blood flow (*P *> 0.05).

## Discussion

Whilst phenotypic (endurance training, heat acclimation) and genotypic differences modulate the level of steady-state skin blood flow and sweating, a significant, albeit modest, amount of that variation during light exercise (trial one) can be explained by an individual's surface-area-to-mass ratio. However, when the work rate was increased (trial two), now forcing a greater reliance upon evaporative cooling, the surface-area-to-mass ratio became a principal determinant of whole-body sweat rate.

## Conclusion

These data indicate that variations in thermoeffector function during steady-state exercise can be explained on the basis of morphological configuration during moderate thermal strain. These observations lead one to hypothesise that heat-related gender differences, and preferential changes in effector function during heat adaptation, might also be explained by differences in body morphology.

